# Semantic embedding based online cross-modal hashing method

**DOI:** 10.1038/s41598-023-50242-w

**Published:** 2024-01-06

**Authors:** Meijia Zhang, Junzheng Li, Xiyuan Zheng

**Affiliations:** 1 School of Data Science and Computer Science, Shandong Women’s University, Jinan, 250300 China; 2 Network Information Management Center, Shandong Management University, Jinan, 250357 China; 3 Shandong Provincial Key Laboratory of Network Based Intelligent Computing, Jinan, 250022 China

**Keywords:** Computational science, Scientific data

## Abstract

Hashing has been extensively utilized in cross-modal retrieval due to its high efficiency in handling large-scale, high-dimensional data. However, most existing cross-modal hashing methods operate as offline learning models, which learn hash codes in a batch-based manner and prove to be inefficient for streaming data. Recently, several online cross-modal hashing methods have been proposed to address the streaming data scenario. Nevertheless, these methods fail to fully leverage the semantic information and accurately optimize hashing in a discrete fashion. As a result, both the accuracy and efficiency of online cross-modal hashing methods are not ideal. To address these issues, this paper introduces the Semantic Embedding-based Online Cross-modal Hashing (SEOCH) method, which integrates semantic information exploitation and online learning into a unified framework. To exploit the semantic information, we map the semantic labels to a latent semantic space and construct a semantic similarity matrix to preserve the similarity between new data and existing data in the Hamming space. Moreover, we employ a discrete optimization strategy to enhance the efficiency of cross-modal retrieval for online hashing. Through extensive experiments on two publicly available multi-label datasets, we demonstrate the superiority of the SEOCH method.

## Introduction

Recently, with the exponential growth of Internet usage, there has been a surge in information data. Traditional single retrieval methods are no longer sufficient to meet the increasing retrieval needs of individuals. Cross-modal retrieval, as a more effective and in-demand search method, has garnered significant research attention in today’s society. Commonly used cross-modal retrieval methods^1–4^ employ real-valued vectors to represent multimodal data. However, these methods require extensive computation and suffer from low efficiency.

To enhance retrieval efficiency, hash-based cross-modal retrieval methods^5–24^ have been proposed. For instance, Asymmetric Supervised Consistent and Specific Hashing (ASCSH)^5^, Fast Discriminative Discrete Hashing (FDDH)^6^, and A Nonlinear Supervised Discrete Hashing (NSDH)^7^, among others. Cross-modal hashing methods can be categorized as unsupervised^11–15^ or supervised^16–19^. In practical applications, supervised hashing methods have shown better performance than unsupervised ones. Despite the progress made in supervised cross-modal hashing research, several challenges remain, such as inadequate exploitation of semantic information, substantial quantization loss, and low retrieval efficiency.

The aforementioned methods all employ an offline learning model for batch-based training, which may fail to adapt to changing data and consequently reduce retrieval efficiency when faced with large volumes of streaming data. To address these limitations, several online hashing methods^25,26^ have been proposed. Similar to offline hashing methods, online hashing methods can also be categorized as unsupervised or supervised. Unsupervised online hashing methods analyze the relationship between sample data, such as dimensionality reduction and the utilization of self-organizing mapping networks. Conversely, supervised online hashing methods often leverage label information to improve retrieval accuracy and mitigate the semantic gap problem.

Although numerous online cross-modal hashing methods have been proposed, existing approaches fail to fully exploit semantic information and accurately optimize hashing in a discrete manner.

To overcome these issues, we propose the Semantic Embedding-based Online Cross-modal Hashing (SEOCH) method, which integrates semantic information exploitation and online learning into a unified framework. To exploit semantic information, we map semantic labels to a latent semantic space and construct a semantic similarity matrix to preserve the similarity between new and existing data in the Hamming space. Moreover, we employ a discrete optimization strategy for online hashing. The main contributions of SEOCH are summarized as follows:

- To exploit semantic information, we map semantic labels to a latent semantic space. Instead of directly projecting semantic labels into binary hash codes *B*, we employ real-valued codes *QB* to leverage supervised information more effectively.
- Subsequently, we construct a semantic similarity matrix to preserve the similarity between new and existing data in the Hamming space, thus mitigating the information loss that occurs when learning hash codes solely based on new data.
- Additionally, we adopt a discrete optimization strategy for online hashing, which reduces quantization errors caused by relaxation-based optimization methods.

The remainder of this paper is organized as follows. We provides an overview of related work in cross-modal hashing methods in the first place. Then, our proposed method and training process are presented. Next, experimental results and corresponding analysis are presented. Finally, we summarize our work.

## Related work

Numerous cross-modal hashing methods have emerged recently. Based on the utilization of semantic label information during the training process, these methods can be categorized into unsupervised and supervised approaches.

Unsupervised methods learn a shared Hamming space without incorporating semantic label information, such as the Inter-Media Hashing (IMH)^27^ method, Collective Matrix Factorization Hashing (CMFH)^13^ method, Fusion Similarity Hashing (FSH)^14^ method, and Latent Semantic Sparse Hashing (LSSH)^12^ method.

On the other hand, supervised hashing methods leverage semantic label information when learning hash codes. Examples include Semantic Correlation Maximization (SCM)^28^, Semantics-Preserving Hashing (SePH)^29^, Discriminant Cross-modal Hashing (DCH)^30^, Subspace Relation Learning for Cross-modal Hashing (SRLCH)^31^, and Semantic Topic Multimodal Hashing (STMH)^32^ method. To take full advantage of heterogeneous correlation, many deep cross-modal retrieval methods have been proposed in recent years, such as references^33–35^. For instance, deep discrete cross-modal hashing with multiple supervision method^34^ designs a semantic network to fully exploit the semantic information implicated in labels, which no longer focuses only on instance-pairwise and class-wise similarities, but also on instance-label level.

The aforementioned methods are all offline cross-modal retrieval models. However, in practical cross-modal retrieval applications, the input is typically in a streaming fashion. Consequently, several online methods have been proposed to cater to this scenario. In the online setting, as new data continuously arrives in a streaming manner, online methods solely utilize the newly arrived data to update the current model. This significantly reduces the computational complexity of the learning algorithm and the storage space requirements. Notable examples include Online Latent Semantic Hashing (OLSH)^25^ and Online Collective Matrix Factorization Hashing (OCMFH)^26^, which have garnered increasing attention. Nevertheless, these methods fail to fully exploit semantic information and accurately optimize hashing in a discrete manner.

## The proposed method

### Notation

In this paper, we consider a scenario where the number of image and text sample is equal. Let $\mathbf{{X}} = \{ {x_i}\} _{i = 1}^n \in {{\mathbb {R}}^{{d_x}*n}}$ represents the image samples and $\mathbf{{Y}} = \{ {y_i}\} _{i = 1}^n \in {{\mathbb {R}}^{{d_y}*n}}$ represents the text samples, where $d_x$ and $d_y$ denote the dimensions of the image and text modalities, respectively, and *n* is the number of samples. ${\varvec{L}} = \left\{ {0,{\hspace{1.0pt}} {\hspace{1.0pt}} {\hspace{1.0pt}} 1{\hspace{1.0pt}} } \right\} \in {{\mathbb {R}}^{c{\hspace{1.0pt}} * {\hspace{1.0pt}} {\hspace{1.0pt}} n}}$ is the label matrix, where *c* is the number of classes. If $$\ \{ {x_i},{\hspace{1.0pt}} {\hspace{1.0pt}} {\hspace{1.0pt}} {y_i}\} \ $$ belongs to the *j*-th class, ${l_{ji}} = 1$, otherwise ${l_{ji}} = 0$. ${\varvec{B}} = \left\{ {0,{\hspace{1.0pt}} {\hspace{1.0pt}} {\hspace{1.0pt}} 1{\hspace{1.0pt}} } \right\} \in {{\mathbb {R}}^{k{\hspace{1.0pt}} * {\hspace{1.0pt}} {\hspace{1.0pt}} n}}$ is the hash code matrix, where *k* represents the number of bits in the hash codes.

Suppose the training data is received in a streaming manner. At the *t*-th round, a new data chunk ${\overrightarrow{X} ^{{{(}}t{{)}}}} \in {{\mathbb {R}}^{{d_x}*{n_t}}}$ or ${\overrightarrow{Y} ^{{{(}}t{{)}}}} \in {{\mathbb {R}}^{{d_y}*{n_t}}}$ with labels ${\overrightarrow{L} ^{{{(}}t{{)}}}} \in {\left\{ {0,1} \right\} ^{c*{n_t}}}$ arrive, where ${n_t}$ denotes the number of new data at *t*-th round. Correspondingly, ${{\tilde{X}}^{{{(}}t{{)}}}} \in {{\mathbb {R}}^{{d_x}*{N_{t - 1}}}}$ or ${{\tilde{Y}}^{{{(}}t{{)}}}} \in {{\mathbb {R}}^{{d_y}*{N_{t - 1}}}}$ with labels ${{\tilde{L}}^{{{(}}t{{)}}}} \in {\left\{ {0,1} \right\} ^{c*{N_{t - 1}}}}$ is the existing data, where ${N_{t - 1}} = \sum \nolimits _{i = 1}^{t - 1} {{n_i}}$ is the number of the existing data before round *t*. The heterogeneous samples ${x_i}$ and ${y_j}$ are associated with similarity matrix *S* with its element ${s_{ij}}$, where ${s_{ij}} = 1$ means ${x_i}$ and ${y_j}$ share at least one common class label, and ${s_{ij}} = 0$ means ${x_i}$ and ${y_j}$ do not share common class label.

### Hash-code learning

To facilitate the online cross modal hashing, the overall objective function (i.e. Loss function ) can be written as:

1 $$ \begin{aligned}   & \mathop {\min }\limits_{\begin{subarray}{l}    Q^{{\left( t \right)}} ,\overrightarrow {B} ^{{\left( t \right)}}  \\    P^{{\left( t \right)}} ,U^{{\left( t \right)}} ,V^{{\left( t \right)}}   \end{subarray} } \lambda _{1} \left\| {Q^{{(t)}} \tilde{B}^{{(t)}}  - P^{{(t)}} \tilde{L}^{{(t)}} } \right\|_{F}^{2}  + \lambda _{2} \left\| {\tilde{B}^{{(t)}}  - U^{{(t)}} \tilde{X}^{{(t)}} } \right\|_{F}^{2}  + \lambda _{3} \left\| {\tilde{B}^{{(t)}}  - V^{{(t)}} \tilde{Y}^{{(t)}} } \right\|_{F}^{2}  + \lambda _{1} \left\| {Q^{{(t)}} \vec{B}^{{(t)}}  - P^{{(t)}} \vec{L}^{{(t)}} } \right\|_{F}^{2}   \hfill \\  &   \quad + \lambda _{2} \left\| {\vec{B}^{{(t)}}  - U^{{(t)}} \vec{X}^{{(t)}} } \right\|_{F}^{2}  + \lambda _{3} \left\| {\vec{B}^{{(t)}}  - V^{{(t)}} \vec{Y}^{{(t)}} } \right\|_{F}^{2}  + \alpha \left( {\left\| {Q^{{(t)}} } \right\|_{F}^{2}  + \left\| {P^{{(t)}} } \right\|_{F}^{2}  + \left\| {U^{{(t)}} } \right\|_{F}^{2}  + \left\| {V^{{(t)}} } \right\|_{F}^{2} } \right) \\ &\quad+ \beta \left\| {\vec{B}^{{(t)T}} \tilde{B}^{{(t)}}  - k\mathop {S^{{(t)}} }\limits^{ \leftrightarrow } } \right\|_{F}^{2}  \hfill \\  \end{aligned}  $$

where $\overset{\leftrightarrow }{S}^{{{(}}t{{)}}}$ is the similarity matrix at round *t*, ${{\tilde{B}}^{{{(}}t{{)}}}} \in {\left\{ {0,1} \right\} ^{k*{N_{t - 1}}}}$ denotes the hash codes of existing data, ${\overrightarrow{B} ^{{{(}}t{{)}}}} \in {\left\{ {0,1} \right\} ^{k*{n_t}}}$ denotes the hash codes of new data. $Q \in {{\mathbb {R}}^{g*k}}$, $P \in {{\mathbb {R}}^{g*c}}$, $U \in {{\mathbb {R}}^{k*{d_x}}}$ and $V \in {{\mathbb {R}}^{k*{d_y}}}$ are four mapping matrices, *g* is the dimension of latent semantic concept space. ${\lambda _1}$, ${\lambda _2}$, ${\lambda _3}$, $\alpha$, $\beta$ are five hyperparameters. The item ${\hspace{1.0pt}} {\hspace{1.0pt}} {\hspace{1.0pt}} \beta {\hspace{1.0pt}} {\hspace{1.0pt}} {\hspace{1.0pt}} \left\| {{{\vec {B}}^{{{(}}t{{)}}T}}{{{\tilde{B}}}^{{{(}}t{{)}}}} - k{{\overset{\leftrightarrow }{S}}^{{{(}}t{{)}}}}} \right\| _F^2{\hspace{1.0pt}} {\hspace{1.0pt}}$ preserves the similarity between the new data and the existing data in the hamming space, which can solve the problem of information loss caused by learning hash codes only with new data.

### Training

The Semantic Embedding based Online Cross-modal Hashing (SEOCH) algorithm aims to optimize five variables. To address the objective in Eq. (1), an alternating learning strategy is employed, updating one variable at a time while keeping the others fixed. The entire training process is outlined below.

#### Update ${\vec {B}^{{{(}}t{{)}}}}$

By fixing all variables except ${\vec {B}^{{{(}}t{{)}}}}$, we can reformulate Eq. (1) as follows:

2 $$\begin{aligned} \begin{array}{l} {\hspace{1.0pt}} {\hspace{1.0pt}} {\hspace{1.0pt}} {\hspace{1.0pt}} \mathop {\min }\limits _{{{\vec {B}}^{{{(}}t{{)}}}}} {\lambda _1}{\hspace{1.0pt}} {\hspace{1.0pt}} \left\| {{Q^{{{(}}t{{)}}}}{{\vec {B}}^{{{(}}t{{)}}}} - {P^{{{(}}t{{)}}}}{{\vec {L}}^{{{(}}t{{)}}}}} \right\| _F^2 + {\lambda _2}\left\| {{{\vec {B}}^{{{(}}t{{)}}}} - {U^{{{(}}t{{)}}}}{{\vec {X}}^{{{(}}t{{)}}}}} \right\| _F^2 + {\lambda _3}\left\| {{{\vec {B}}^{{{(}}t{{)}}}} - {V^{{{(}}t{{)}}}}{{\vec {Y}}^{{{(}}t{{)}}}}} \right\| _F^2{\hspace{1.0pt}} {\hspace{1.0pt}} + \beta \left\| {{{\vec {B}}^{{{(}}t{{)}}T}}{{{\tilde{B}}}^{{{(}}t{{)}}}} - k{{\overset{\leftrightarrow }{S}}^{{{(}}t{{)}}}}} \right\| _F^2{\hspace{1.0pt}} {\hspace{1.0pt}} \end{array} \end{aligned}$$

Differentiating Eq. (2) with respect to ${\vec {B}^{{{(}}t{{)}}}}$ and setting it to zero, we obtain:

3 $$\begin{aligned} \begin{array}{l} {{\vec {B}}^{{{(}}t{{)}}}} = {\hspace{1.0pt}} {{{(}}{\lambda _1}{Q^{{{(}}t{{)}}T}}{Q^{{{(}}t{{)}}}}{\hspace{1.0pt}} + {\lambda _2}{I_1} + {\lambda _3}{I_1}\mathrm{{ + }}\beta {{{\tilde{B}}}^{{{(}}t{{)}}}}{{{\tilde{B}}}^{{{(}}t{{)}}T}}{{)}}^{ - 1}}{\hspace{1.0pt}} * {{(}}{\lambda _3}{V^{{{(}}t{{)}}}}{{\vec {Y}}^{{{(}}t{{)}}}} + {\lambda _2}{U^{{{(}}t{{)}}}}{{\vec {X}}^{{{(}}t{{)}}}} + {\lambda _1}{Q^{{{(}}t{{)}}T}}{P^{{{(}}t{{)}}}}{{\vec {L}}^{{{(}}t{{)}}}} + \beta {{{\tilde{B}}}^{{{(}}t{{)}}}}k{{\overset{\leftrightarrow }{S}}^{{{(}}t{{)}}T}}{{)}} \end{array} \end{aligned}$$

where ${I_1} \in {{\mathbb {R}}^{k*k}}$ denotes an identity matrix. To compute ${{\tilde{B}}^{{{(}}t{{)}}}}$ in Eq. (3), we follow the steps below.

By fixing all variables except ${{\tilde{B}}^{{{(}}t{{)}}}}$, we can reformulate Eq. (1) as follows:

4 $$\begin{aligned} \begin{array}{l} \mathop {\min }\limits _{{{{\tilde{B}}}^{{{(}}t{{)}}}}} {\hspace{1.0pt}} {\hspace{1.0pt}} {\hspace{1.0pt}} {\lambda _1}{\hspace{1.0pt}} {\hspace{1.0pt}} {\hspace{1.0pt}} \left\| {{Q^{{{(}}t{{)}}}}{{{\tilde{B}}}^{{{(}}t{{)}}}} - {P^{{{(}}t{{)}}}}{{{\tilde{L}}}^{{{(}}t{{)}}}}} \right\| _F^2 + {\lambda _2}\left\| {{{{\tilde{B}}}^{{{(}}t{{)}}}} - {U^{{{(}}t{{)}}}}{{{\tilde{X}}}^{{{(}}t{{)}}}}} \right\| _F^2 + {\lambda _3}\left\| {{{{\tilde{B}}}^{{{(}}t{{)}}}} - {V^{{{(}}t{{)}}}}{{{\tilde{Y}}}^{{{(}}t{{)}}}}} \right\| _F^2{\hspace{1.0pt}} {\hspace{1.0pt}} {\hspace{1.0pt}} {\hspace{1.0pt}} + \beta {\left\| {{{\vec {B}}^{{{(}}t{{)}}T}}{{{\tilde{B}}}^{{{(}}t{{)}}}} - k{{\overset{\leftrightarrow }{S}}^{{{(}}t{{)}}}}} \right\| _F^2}{\hspace{1.0pt}} {\hspace{1.0pt}} \end{array} \end{aligned}$$

Differentiating Eq. (4) with respect to ${{\tilde{B}}^{{{(}}t{{)}}}}$ and setting it to zero, we obtain:

5 $$\begin{aligned} {\tilde{B}}^{(t)} = {\textrm{sgn}} (\beta {\vec {B}}^{(t)} \overset{\leftrightarrow }{S}^{(t)}) + (\lambda _{1} {Q^{(t)}}^{T} Q^{(t)} + \lambda _{2} I_{1} + \lambda _{3} I_{1})^{- 1} * (\lambda _{1} {Q^{(t)}}^{T} P^{(t)} {\tilde{L}}^{(t)} + \lambda _{2} U^{(t)} {\tilde{X}}^{(t)} + \lambda _{3} V^{(t)} {\tilde{Y}}^{(t)}) \end{aligned}$$

where ${I_1} \in {{\mathbb {R}}^{k*k}}$ denotes an identity matrix.

#### Update ${Q^{{{(}}t{{)}}}}$

By differentiating Eq. (1) with respect to ${Q^{{{(}}t{{)}}}}$,

6 $$ \frac{{\partial Loss}}{{\partial Q^{{(t)}} }} = 2\lambda _{1} (Q^{{(t)}} \tilde{B}^{{(t)}}  - P^{{(t)}} \tilde{L}^{{(t)}} )\tilde{B}^{{(t)T}}  + 2\lambda _{1} (Q^{{(t)}} \vec{B}^{{(t)}}  - P^{{(t)}} \vec{L}^{{(t)}} )\vec{B}^{{(t)T}}  + 2\alpha Q^{{(t)}}  $$

By setting Eq. (6) to zero, we have

7 $$ Q^{{(t)}}  = C_{2}^{{(t)}} \left( {C_{1}^{{(t)}} {\text{ + }}\frac{\alpha }{{\lambda _{1} }}I_{1} } \right)^{{ - 1}}  $$

where

8 $$ \begin{array}{*{20}l}    {C_{1}^{{(t - 1)}}  = \tilde{B}^{{(t)}} \tilde{B}^{{(t)T}}  + \vec{B}^{{(t)}} \vec{B}^{{(t)T}} } \hfill  \\    {C_{2}^{{(t - 1)}}  = P^{{(t)}} \tilde{L}^{{(t)}} \tilde{B}^{{(t)T}}  + P^{{(t)}} \vec{L}^{{(t)}} \vec{B}^{{(t)T}} } \hfill  \\   \end{array}  $$

9 $$ \begin{array}{*{20}l}    {C_{1}^{{(t)}}  = C_{1}^{{(t - 1)}}  + \vec{B}^{{(t)}} \vec{B}^{{(t)T}} } \hfill  \\    {C_{2}^{{(t)}}  = C_{2}^{{(t - 1)}}  + P^{{(t)}} \vec{L}^{{(t)}} \vec{B}^{{(t)T}} } \hfill  \\   \end{array}  $$

#### Update ${P^{{{(}}t{{)}}}}$

By differentiating Eq. (1) with respect to ${P^{{{(}}t{{)}}}}$,

10 $$\begin{aligned} \small \frac{{\partial Loss}}{{\partial {P^{{{(}}t{{)}}}}}} = {\hspace{1.0pt}} {\hspace{1.0pt}} {\hspace{1.0pt}} {\hspace{1.0pt}} 2{\lambda _1}{{(}}{Q^{{{(}}t{{)}}}}{{\tilde{B}}^{{{(}}t{{)}}}} - {P^{{{(}}t{{)}}}}{{\tilde{L}}^{{{(}}t{{)}}}}{{)(}} - {{\tilde{L}}^{{{(}}t{{)}}T}}{{)}} + 2{\lambda _1}{{(}}{Q^{{{(}}t{{)}}}}{\vec {B}^{{{(}}t{{)}}}} - {P^{{{(}}t{{)}}}}{\vec {L}^{{{(}}t{{)}}}}{{)(}} - {\vec {L}^{{{(}}t{{)}}T}}{{) + 2}}\alpha {P^{{{(}}t{{)}}}} \end{aligned}$$

By setting Eq. (10) to zero, we have

11 $$\begin{aligned} {P^{{{(}}t{{)}}}} = D_2^{{{(}}t{{)}}}{{{(}}D_1^{{{(}}t{{)}}}\mathrm{{ + }}\frac{\alpha }{{{\lambda _1}}}{I_2}{{)}}^{ - 1}} \end{aligned}$$

where ${I_2} \in {{\mathbb {R}}^{c*c}}$ is an identity matrix,

12 $$\begin{aligned}{} & {} \begin{array}{l} D_1^{{{(}}t{{)}}} = {{{\tilde{L}}}^{{{(}}t{{)}}}}{{{\tilde{L}}}^{{{(}}t{{)}}T}} + {{\vec {L}}^{{{(}}t{{)}}}}{{\vec {L}}^{{{(}}t{{)}}T}}\\ D_2^{{{(}}t{{)}}} = {Q^{{{(}}t{{)}}}}{{{\tilde{B}}}^{{{(}}t{{)}}}}{{{\tilde{L}}}^{{{(}}t{{)}}T}} + {Q^{{{(}}t{{)}}}}{{\vec {B}}^{{{(}}t{{)}}}}{{\vec {L}}^{{{(}}t{{)}}T}} \end{array} \end{aligned}$$

13 $$\begin{aligned}{} & {} \begin{array}{l} D_1^{{{(}}t{{)}}} = D_1^{{{(}}t - 1{{)}}} + {{\vec {L}}^{{{(}}t{{)}}}}{{\vec {L}}^{{{(}}t{{)}}T}}\\ D_2^{{{(}}t{{)}}} = D_2^{{{(}}t - 1{{)}}} + {Q^{{{(}}t{{)}}}}{{\vec {B}}^{{{(}}t{{)}}}}{{\vec {L}}^{{{(}}t{{)}}T}} \end{array} \end{aligned}$$

#### Update ${U^{{{(}}t{{)}}}}$

By differentiating Eq. (1) with respect to ${U^{{{(}}t{{)}}}}$ ,

14 $$\begin{aligned} \frac{{\partial Loss}}{{\partial {U^{{{(}}t{{)}}}}}}{\hspace{1.0pt}} {\hspace{1.0pt}} {\hspace{1.0pt}} {\hspace{1.0pt}} {\hspace{1.0pt}} {\hspace{1.0pt}} = 2{\lambda _2}{{(}}{{\tilde{B}}^{{{(}}t{{)}}}} - {U^{{{(}}t{{)}}}}{{\tilde{X}}^{{{(}}t{{)}}}}{{)(}} - {{\tilde{X}}^{{{(}}t{{)}}T}}{{)}} + {\hspace{1.0pt}} 2{\lambda _2}{{(}}{\vec {B}^{{{(}}t{{)}}}} - {U^{{{(}}t{{)}}}}{\vec {X}^{{{(}}t{{)}}}}{{)(}} - {\vec {X}^{{{(}}t{{)}}T}}{{) + 2}}\alpha {U^{{{(}}t{{)}}}} \end{aligned}$$

By setting Eq. (14) to zero, we have

15 $$\begin{aligned} {U^{{{(}}t{{)}}}} = E_2^{{{(}}t{{)}}} \cdot {{{(}}E_1^{{{(}}t{{)}}}\mathrm{{ + }}\frac{\alpha }{{{\lambda _2}}}{I_3}{{)}}^{ - 1}} \end{aligned}$$

where ${I_3} \in {{\mathbb {R}}^{{d_x}*{d_x}}}$ is an identity matrix,

16 $$\begin{aligned}{} & {} \begin{array}{l} E_1^{{{(}}t - 1{{)}}} = {{{\tilde{X}}}^{{{(}}t{{)}}}}{{{\tilde{X}}}^{{{(}}t{{)}}T}} + {{\vec {X}}^{{{(}}t{{)}}}}{{\vec {X}}^{{{(}}t{{)}}T}}\\ E_2^{{{(}}t - 1{{)}}} = {{{\tilde{B}}}^{{{(}}t{{)}}}}{{{\tilde{X}}}^{{{(}}t{{)}}T}}{\hspace{1.0pt}} + {\hspace{1.0pt}} {{\vec {B}}^{{{(}}t{{)}}}}{{\vec {X}}^{{{(}}t{{)}}T}} \end{array} \end{aligned}$$

17 $$\begin{aligned}{} & {} \begin{array}{l} E_1^{{{(}}t{{)}}} = E_1^{{{(}}t - 1{{)}}} + {{\vec {X}}^{{{(}}t{{)}}}}{{\vec {X}}^{{{(}}t{{)}}T}}\\ E_2^{{{(}}t{{)}}} = E_2^{{{(}}t - 1{{)}}}{\hspace{1.0pt}} + {\hspace{1.0pt}} {{\vec {B}}^{{{(}}t{{)}}}}{{\vec {X}}^{{{(}}t{{)}}T}} \end{array} \end{aligned}$$

#### Update ${V^{{{(}}t{{)}}}}$

By differentiating Eq. (1) with respect to ${V^{{{(}}t{{)}}}}$,

18 $$\begin{aligned} \frac{{\partial Loss}}{{\partial {V^{{{(}}t{{)}}}}}}{\hspace{1.0pt}} {\hspace{1.0pt}} {\hspace{1.0pt}} = 2{\lambda _3}{{(}}{{\tilde{B}}^{{{(}}t{{)}}}} - {V^{{{(}}t{{)}}}}{{\tilde{Y}}^{{{(}}t{{)}}}}{{)}}{\hspace{1.0pt}} {\hspace{1.0pt}} {\hspace{1.0pt}} {{(}} - {{\tilde{Y}}^{{{(}}t{{)}}T}}{{)}}{\hspace{1.0pt}} {\hspace{1.0pt}} + 2{\lambda _3}{{(}}{\vec {B}^{{{(}}t{{)}}}} - {V^{{{(}}t{{)}}}}{\vec {Y}^{{{(}}t{{)}}}}{{)}}{\hspace{1.0pt}} {\hspace{1.0pt}} {{(}} - {\vec {Y}^{{{(}}t{{)}}T}}{{)}}{\hspace{1.0pt}} {\hspace{1.0pt}} \mathrm{{ + 2}}\alpha {V^{{{(}}t{{)}}}} \end{aligned}$$

By setting Eq. (18) to zero, we have

19 $$\begin{aligned}{} & {} {V^{{{(}}t{{)}}}} = F_2^{{{(}}t{{)}}} \cdot {{{(}}F_1^{{{(}}t{{)}}}\mathrm{{ + }}\frac{\alpha }{{{\lambda _3}}}{I_4}{{)}}^{ - 1}} \end{aligned}$$

20 $$\begin{aligned}{} & {} \begin{array}{l} F_1^{{{(}}t - 1{{)}}} = {{{\tilde{Y}}}^{{{(}}t{{)}}}}{{{\tilde{Y}}}^{{{(}}t{{)}}T}} + {{\vec {Y}}^{{{(}}t{{)}}}}{{\vec {Y}}^{{{(}}t{{)}}T}}\\ F_2^{{{(}}t - 1{{)}}} = {{{\tilde{B}}}^{{{(}}t{{)}}}}{{{\tilde{Y}}}^{{{(}}t{{)}}T}} + {\hspace{1.0pt}} {{\vec {B}}^{{{(}}t{{)}}}}{{\vec {Y}}^{{{(}}t{{)}}T}} \end{array} \end{aligned}$$

21 $$\begin{aligned}{} & {} \begin{array}{l} F_1^{{{(}}t{{)}}} = F_1^{{{(}}t - 1{{)}}} + {{\vec {Y}}^{{{(}}t{{)}}}}{{\vec {Y}}^{{{(}}t{{)}}T}}\\ F_2^{{{(}}t{{)}}} = F_2^{{{(}}t - 1{{)}}}{\hspace{1.0pt}} + {\hspace{1.0pt}} {{\vec {B}}^{{{(}}t{{)}}}}{{\vec {Y}}^{{{(}}t{{)}}T}} \end{array} \end{aligned}$$

where ${I_4} \in {{\mathbb {R}}^{{d_y}*{d_y}}}$ is an identity matrix.

### Out of sample

For a query that is not in the training set, we can generate the hash codes of a query point ${x_q}$ or ${y_q}$ as follows,

22 $$\begin{aligned} {{\textrm{b}}_q} = {\textrm{sign}}({x_q}{U^{{{(}}t{{)}}}}{{),}}{\hspace{1.0pt}} {\hspace{1.0pt}} {\hspace{1.0pt}} {\hspace{1.0pt}} {\hspace{1.0pt}} {\hspace{1.0pt}} {\hspace{1.0pt}} {{\textrm{b}}_q} = {\textrm{sign}}({y_q}{V^{{{(}}t{{)}}}}{{)}} \end{aligned}$$

To obtain a comprehensive overview, the complete learning algorithm of our proposed SEOCH is presented in Algorithm 1.

**Algorithm 1:**
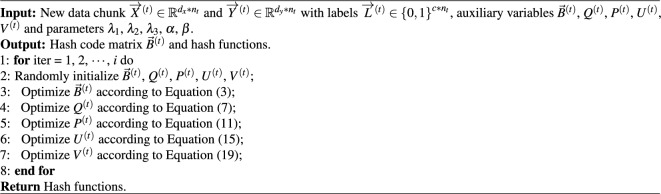
The comprehensive learning algorithm of our proposed SEOCH

## Experiments

### Datasets

In order to thoroughly assess the effectiveness of our approach, we conduct experiments on two publicly available multi-label datasets, namely the **MIRFLICKR-25K** dataset and the **NUS-WIDE** dataset. Detailed descriptions of these datasets are provided below.

The **MIRFLICKR-25K** dataset comprises 25,000 images with a total of 24 labels. Each image in this dataset is associated with one or more labels and connected to several textual tags. From this dataset, we randomly select 20,015 image-text pairs that possess at least 20 textual tags. Among these pairs, 2000 are chosen as queries and the remaining pairs form the training set. The image and text features used are 512-dimensional Scale-Invariant Feature Transform (SIFT) features and 1386-dimensional Bag of Words (BoW) features, respectively. To facilitate online cross-modal hashing, the training set is divided into 9 data chunks, with the first 8 chunks containing 2,000 instances each and the last chunk containing 2015 instances.

The **NUS-WIDE** dataset consists of approximately 270,000 images annotated with a total of 81 labels. For our experiments, we select 186,577 image-text pairs that are associated with at least one of the 10 most frequent concepts. Within the NUS-WIDE dataset, we randomly choose 1,867 pairs as queries, while the remaining pairs serve as the database. The image and text features in the database are represented by 500-dimensional Bag-of-Visual Words (BoVW) features and 1000-dimensional BoW features, respectively. Similar to the previous dataset, the training set is divided into 18 data chunks, with the first 17 chunks containing 10,000 instances each and the last chunk containing 14,710 instances to facilitate online cross-modal hashing.

### Baselines and evaluated metrics

The proposed method is evaluated against six state-of-the-art cross-modal hashing methods, which can be categorized as follows: (1) offline methods: SCM-seq^28^, DCH^30^, SRLCH^31^, JIMFH^36^; (2) online methods: OLSH^25^, OCMFH^26^. The source codes of these baselines are publicly available online, and the parameters are set based on the recommendations provided in the corresponding papers. In JIMFH, the mAP value is calculated with the number of query data set to 100. To ensure a fair comparison, we set the number of query data to 2,000 and 1,867 for the MIRFLICKR-25K and NUS-WIDE datasets, respectively.

Consistent with previous studies, we employ mean Average Precision (mAP) and Precision-Recall curves to evaluate the retrieval accuracy for two retrieval tasks: Image Retrieval Text (I2T) and Text Retrieval Image (T2I).

In the experiments, the parameters are set empirically. For the MIRFLICKR-25K dataset, we set ${\lambda _1}$ =1e3, ${\lambda _2}$=0.1, ${\lambda _3}$=1, $\alpha$=0.8, $\beta$=1, and *g*=50. For the NUS-WIDE dataset, we set ${\lambda _1}$ =1e4, ${\lambda _2}$ =0.1, ${\lambda _3}$ =0.1, $\alpha$=0.1, $\beta$ =0.1, and *g*=100.

### Experimental results and analysis

**Figure 1:**
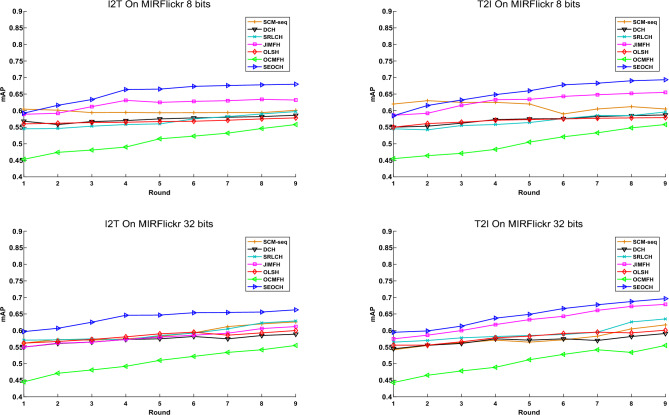
The mAP scores at each round for two retrieval tasks on the **MIRFLICKR-25K** dataset.

**Figure 2:**
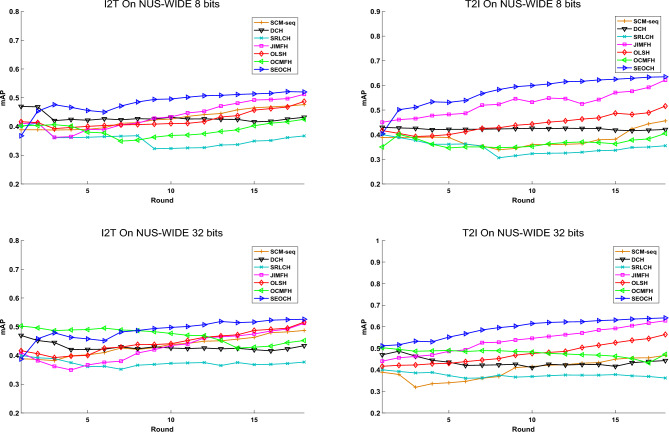
The mAP scores at each round for two retrieval tasks on the **NUS-WIDE** dataset.

The mean Average Precision (mAP) scores of SEOCH and the comparison methods in the final round on the MIRFLICKR-25K and NUS-WIDE datasets are presented in Tables 1 and 2, respectively. Moreover, Figs. 1 and 2 display the mAP scores for each round of different methods in the two datasets, using 8-bit and 32-bit hash codes.

**Table 1:** The mAP scores of SEOCH and the comparison methods in the final round on the **MIRFLICKR-25K** dataset (Numbers in boldface indicate the highest scores).

Task	Methods	code length				
		8 bits	16 bits	32bits	64 bits	128 bits
Image to Text	SCM-Seq	0.601	0.616	0.626	0.632	**0.634**
	DCH	0.586	0.59	0.589	0.609	0.623
	SRLCH	0.597	0.604	0.629	0.614	0.627
	JIMFH	0.632	0.636	0.612	0.637	0.626
	OLSH	0.578	0.577	0.60	0.595	0.593
	OCMFH	0.558	0.556	0.555	0.555	0.554
	SEOCH	**0.68**	**0.645**	**0.663**	**0.64**	0.62
Text to Image	SCM-Seq	0.605	0.611	0.617	0.62	0.622
	DCH	0.587	0.592	0.591	0.604	0.618
	SRLCH	0.596	0.605	0.635	0.619	0.633
	JIMFH	0.655	0.659	0.679	0.679	**0.69**
	OLSH	0.579	0.582	0.601	0.602	0.602
	OCMFH	0.558	0.556	0.555	0.555	0.554
	SEOCH	**0.693**	**0.695**	**0.696**	**0.68**	0.66

**Table 2:** The mAP scores of SEOCH and the comparison methods in the final round on the **NUS-WIDE** dataset (Numbers in boldface indicate the highest scores).

Task	Methods	code length				
		8 bits	16 bits	32bits	64 bits	128 bits
Image to Text	SCM-Seq	0.476	0.479	0.487	0.486	0.491
	DCH	0.432	0.434	0.453	0.493	0.504
	SRLCH	0.367	0.377	0.347	0.352	0.365
	JIMFH	0.512	0.512	0.515	0.518	0.52
	OLSH	0.487	0.516	0.524	0.535	0.526
	OCMFH	0.425	0.452	0.484	0.465	0.45
	SEOCH	**0.525**	**0.526**	**0.535**	**0.536**	**0.532**
Text to Image	SCM-Seq	0.456	0.459	0.467	0.468	0.471
	DCH	0.421	0.424	0.443	0.479	0.487
	SRLCH	0.355	0.356	0.362	0.365	0.364
	JIMFH	0.622	0.624	0.628	0.623	0.62
	OLSH	0.516	0.559	0.564	0.592	0.58
	OCMFH	0.405	0.438	0.472	0.455	0.441
	SEOCH	**0.635**	**0.636**	**0.641**	**0.642**	**0.638**

From the above results, it can be observed that: (1) The proposed SEOCH significantly outperforms all the offline baselines in almost all tasks, demonstrating its efficiency for streaming data scenarios. (2) The SEOCH outperforms the online baselines in most of the retrieval tasks, indicating the superiority of the semantic embedding-based learning method. (3) The discrete methods, namely JIMFH, significantly outperform the relaxation-based methods, i.e., SCM-seq, validating that the discrete hashing methods are more effective for semantic similarity preservation. (4) With the increase of the code length, the performance of all methods is improved, which is consistent with the general observation in hashing research. (5) Compared with the 8-bit and 16-bit hash codes, the performance improvement of SEOCH is more significant when using longer codes (e.g., 32 bits or above), indicating its ability to exploit the semantic structure of the data in high-dimensional Hamming space.

### Further analysis

#### Ablation study

Moreover, three variations of SEOCH have been devised to assess the performance of the proposed method, as presented in Table 3. SEOCH-I sets ${\lambda _1}$ to 0. SEOCH-II sets ${\lambda _2}$ and ${\lambda _3}$ to 0. SEOCH -III eliminates the similarity matrix. From Table 3, it can be observed that for 8 bits, SEOCH-III achieves the lowest result; for 16, 32, and 64 bits, SEOCH-II exhibits the lowest result; for 128 bits, SEOCH-I demonstrates the lowest result. Hence, it can be concluded that each component in our proposed SEOCH plays a significant role in the retrieval outcomes.

**Table 3:** Ablation study on the **MIRFLICKR-25K** dataset (The numbers in bold indicate the best performance).

Task	Methods	code length				
		8 bits	16 bits	32bits	64 bits	128 bits
Image to Text	SEOCH-I	0.609	0.607	0.587	0.571	0.561
	SEOCH-II	0.64	0.554	0.579	0.549	0.568
	SEOCH-III	0.577	0.605	0.612	0.635	0.612
	SEOCH	**0.68**	**0.645**	**0.663**	**0.64**	**0.62**
Text to Image	SEOCH-I	0.623	0.619	0.602	0.581	0.566
	SEOCH-II	0.667	0.578	0.598	0.59	0.577
	SEOCH-III	0.575	0.617	0.632	0.649	0.658
	SEOCH	**0.693**	**0.695**	**0.696**	**0.68**	**0.66**

#### Time cost analysis

To validate the efficiency of the proposed SEOCH, we conducted additional experiments on the MIRFLICKR-25K dataset to compare the training times of the baseline methods and SEOCH. In these experiments, we configured the hash code length to be 8 bits and 32 bits respectively. The training times of the two online methods under the same configurations are presented in Table 4.

**Table 4:** Training time (in seconds) on the **MIRFLICKR-25K** dataset.

Bits	Methods	Chunk1	Chunk2	Chunk3	Chunk4	Chunk5
8	OLSH	4.6	1.34	1.32	1.32	1.33
	OCMFH	13.35	1.52	1.36	1.43	1.49
	SEOCH	2.01	0.64	1.75	1.03	1.19
32	OLSH	5.4	1.41	1.42	1.52	1.53
	OCMFH	15.74	1.43	1.34	1.93	1.79
	SEOCH	2.42	0.82	1.93	1.37	1.9

From Table 4, it is evident that the proposed SEOCH not only achieves superior retrieval performance but also exhibits the shortest training time. Hence, the retrieval efficiency has been significantly enhanced.

## Conclusion

This paper is focused on harnessing the semantic correlation between different modalities and enhancing the efficiency of cross-modal retrieval in online scenarios. In this paper, we propose an innovative approach called Semantic Embedding based Online Cross-modal Hashing (SEOCH). SEOCH integrates the exploitation of semantic information and online learning into a unified framework. To leverage semantic information, we map semantic labels to a latent semantic space and construct a semantic similarity matrix to preserve the similarity between new and existing data in the Hamming space. Moreover, we employ a discrete optimization strategy for online hashing. Extensive experiments on two publicly available multi-label datasets validate the superiority of SEOCH.

## Data Availability

The datasets generated and/or analysed during the current study can be accessed as follows: Download the [NUSWIDE.mat] dataset from https://pan.baidu.com/s/1WEAezxn6mbEbqekPjBnRQw, password: 8888. Download the [MIRFLICKR.mat] dataset from https://pan.baidu.com/s/1GT-mrUutslGhp3lP2i_rYQ, password: 8888. The source code of Semantic embedding based online cross-modal hashing method are available from the corresponding author on reasonable request.
